# Individual and combined associations between cardiorespiratory fitness and grip strength with common mental disorders: a prospective cohort study in the UK Biobank

**DOI:** 10.1186/s12916-020-01782-9

**Published:** 2020-11-11

**Authors:** Aaron A. Kandola, David P. J. Osborn, Brendon Stubbs, Karmel W. Choi, Joseph F. Hayes

**Affiliations:** 1 Division of Psychiatry, University College London, Maple House, 149 Tottenham Court Rd, London, W1T 7BN UK; 2 Camden and Islington NHS Foundation Trust, London, UK; 3 Department of Psychological Medicine, Institute of Psychiatry, Psychology, and Neuroscience, King’s College London, London, UK; 4 Physiotherapy Department, South London, and Maudsley National Health Services Foundation Trust, London, UK; 5 Department of Psychiatry, Massachusetts General Hospital, Boston, MA USA; 6 Department of Epidemiology, Harvard T.H. Chan School of Public Health, Boston, MA USA; 7 Psychiatric & Neurodevelopmental Genetics Unit, Center for Genomic Medicine, Massachusetts General Hospital, Boston, MA USA; 8 Stanley Center for Psychiatric Research, Broad Institute, Boston, MA USA

**Keywords:** Fitness, Depression, Anxiety, Physical activity, Exercise, Cardiorespiratory, Grip strength, Muscular, Prevention

## Abstract

**Background:**

Depression and anxiety are common mental disorders that increase physical health risks and are leading causes of global disability. Several forms of physical fitness could be modifiable risk factors for common mental disorders in the population. We examined associations between individual and combined markers of cardiorespiratory fitness and grip strength with the incidence of common mental disorders.

**Methods:**

A 7-year prospective cohort study in 152,978 UK Biobank participants. An exercise test and dynamometer were used to measure cardiorespiratory and grip strength, respectively. We used Patient Health Questionnaire-9 and Generalised Anxiety Disorder-7 scales to estimate the incidence of common mental disorders at follow-up.

**Results:**

Fully adjusted, longitudinal models indicated a dose-response relationship. Low and medium cardiorespiratory fitness was associated with 1.485 (95% CIs, 1.301 to 1.694, *p* <  0.001) and 1.141 (95% CIs, 1.005 to 1.297, *p* = 0.041) higher odds of depression or anxiety, compared to high cardiorespiratory fitness. Low and medium grip strength was associated with 1.381 (95% CIs, 1.315 to 1.452, *p* <  0.001) and 1.116 (95% CIs, 1.063 to 1.172, *p* <  0.001) higher odds of common mental disorder compared to high grip strength.

Individuals in the lowest group for both cardiorespiratory fitness and grip strength had 1.981 (95% CIs, 1.553 to 2.527, *p* <  0.001) higher odds of depression, 1.599 (95% CIs, 1.148 to 2.118, *p* = 0.004) higher odds of anxiety, and 1.814 (95% CIs, 1.461 to 2.252, *p* <  0.001) higher odds of either common mental disorder, compared to high for both types of fitness.

**Conclusions:**

Objective cardiorespiratory and muscular fitness markers represent modifiable risk factors for common mental disorders. Public health strategies to reduce common mental disorders could include combinations of aerobic and resistance activities.

## Background

Common mental disorders are major contributors to the global health burden, with depression and anxiety disorders being the first and sixth leading causes of disability worldwide, respectively [1]. They can substantially affect daily functioning and are associated with elevated physical health risks over time, including a higher incidence of cardiovascular disease and premature mortality [2–4]. Structured physical activity interventions have been shown to reduce common mental health symptoms with a moderate-to-large effect size [5–11]. Low physical activity may be a modifiable, population-level risk factor for common mental disorders [12–16]. However, nearly prior all studies use self-reported measures of activity, which are prone to bias [17]. Physical activity has a major influence on cardiorespiratory fitness (CRF) and muscular strength [18, 19], two related but distinct markers of physical fitness that are reliable indicators of overall health, disease risk, and mortality [20–27]. Both are measurable in large groups through validated fitness tests that produce objective outputs, which may also act as surrogate markers of habitual physical activity that are not reliant on self-report [18, 28].

There is limited research on the associations between physical fitness markers and the incidence of common mental disorders. A recent meta-analysis of prospective cohort studies found that low CRF was associated with a 47% (HR = 1.47, 95% CI 1.23, 1.76) higher incidence rate of common mental disorders [28]. However, these results were from a small number of studies (*n* = 4) with substantial heterogeneity (*I*^2^ = 85.1%) and most focused on depression rather than anxiety. Fewer studies have focused on the role of overall muscular strength, for which grip strength is a simple clinical proxy measure [29–31]. Some small cross-sectional [32, 33] and longitudinal [34–36] studies suggest that low grip strength is associated with a higher incidence of depression and one with anxiety [37]. However, longitudinal findings have been inconsistent, and more high-quality data from large samples are necessary. Most studies only focus on depression, despite anxiety disorders being another major source of global disability and highly comorbid with depression [38].

Low physical fitness, indexed by CRF or grip strength, could be a useful risk factor for common mental disorders in the population. We are not aware of any large-scale studies focusing on associations between individual and combined CRF and grip strength with the incidence of common mental disorders. CRF and grip strength reflect different physiological profiles and indicate different types of habitual physical activity, i.e. aerobic vs. resistance training. Combined training to increase both CRF and strength leads to improved physical health outcomes than focusing on either component of fitness alone [39–42] and the same may be true for mental health. A recent cross-sectional study in adolescents found that CRF and not muscular strength was associated with fewer psychological difficulties, but did not assess the combination of CRF and muscular strength [43].

To address these knowledge gaps, we conducted the first prospective study to examine associations between individual and combined markers of CRF and grip strength with the incidence of depression and anxiety. We aimed to (1) examine longitudinal associations between CRF, grip strength, and the incidence of common mental disorders and (2) investigate associations between combined fitness levels with the incidence of common mental disorders.

## Methods

### Participants

The UK Biobank is a prospective cohort study that recruited 502,682 participants (5.5% response rate) aged 40 to 69 years from the general population of England, Scotland, and Wales, between April 2007 and December 2010 [44]. Participants completed a baseline battery of touchscreen questionnaires, physical measures, imaging, genetic, and biological assessments in 22 research centres across the UK [45]. Our sample included participants who had a valid measure of baseline symptoms and at least one measure of grip strength or CRF (*n* = 491,278) at baseline. For the longitudinal analyses, we restricted the sample to participants who also had a completed Patient Health Questionnaire-9 (PHQ-9) and Generalised Anxiety Disorder-7 (GAD-7) at follow-up (2017) and at least one measure of grip strength or CRF (*n* = 152,978).

### Exposure(s): cardiorespiratory fitness and grip strength

A subset of Biobank participants completed fitness tests between August 2009 and December 2010. Participants completed a 6-min submaximal exercise test on a stationary bike (eBike Comfort Ergometer, General Electric). Individualised protocols were developed to calculate an appropriate workload based on participants’ age, height, weight, sex, and resting heart rate. A four-lead ECG was used to monitor heart rate before, during, and in the recovery phase following the test.

We followed protocols from previous studies to estimate CRF from the submaximal tests [46, 47]. We first estimated the work rate participants would have achieved in a maximal fitness test. The maximal work rate maps onto maximum oxygen consumption (VO_2max_), which is an indicator of CRF. We estimated maximal work rate (measured in watts) from heart rate before the test, maximum heart rate during the test, and work rate at the end of the test using linear regression. We then extrapolated the regression line to participants’ age-predicted maximal heart rate using the equation: 208 – 7 × age [48], assuming a linear relationship.

To estimate maximum oxygen consumption, we used the equation: 7 + (10.8 × maximal work rate (in watts))/body weight (in kg)) [49]. Maximal oxygen consumption is a continuous measure of CRF expressed in millilitres of oxygen per kilogramme of body weight per minute (ml kg-1.min-1). We used metabolic equivalents (METs) to express CRF output, where 1 MET is 3.5 ml kg-1.min-1.

Grip strength was measured using a Jamar j00105 hydraulic hand dynamometer in each hand. Participants grasp the handle sitting in an upright position and squeeze as strongly as possible for 3 s. The output was expressed in kilogrammes (kg), taking the mean value of each hand.

For the main analysis, we created age- and sex-adjusted tertiles of grip strength and CRF, in line with previous work [47]. The tertiles represent low, medium, and high fitness groups. These groupings were to aid interpretation and account for possible non-linear associations between fitness and mental health. We also used continuous CRF and grip strength exposure variables in the secondary analysis, with CRF presented in METs and grip strength in 5 kg increments as in previous studies [46].

### Outcome(s): common mental disorders

At baseline (2006 to 2010), common mental health symptoms were measured using 3 questions from Patient Health Questionnaire-9 (PHQ-9) that cover core features of depression (low mood, anhedonia, and lethargy) [50]. It contains an additional question adapted from the PHQ-9 to cover tenseness, a common feature of anxiety disorders. The questionnaire uses a four-point ordinal scale from 0 (not at all) to 3 (nearly every day), with scores ranging from 0 to 12. Ultra-brief adaptations of the PHQ-9 have good agreement with longer scales for both depression and anxiety symptoms [51]. We used a continuous symptom score for this measure due to the lack of a valid cut-off.

At follow-up, common mental health symptoms were measured in a 2017 Mental Health Questionnaire, which included the PHQ-9 and Generalised Anxiety Disorder-7 (GAD-7) questionnaire. The PHQ-9 is a depression screening instrument with nine questions on a four-point ordinal scale from 0 (not at all) to 3 (nearly every day) [50]. Total scores range from 0 to 27, with higher scores indicating greater symptom severity. We defined possible incidences of depression using an established cut-off (scores ≥ 10) for the main analysis and a continuous symptom score (secondary analysis). Previous studies show that a cut-off score of ≥ 10 has 88% sensitivity and specificity for identifying incident major depression [50].

The GAD-7 is a 7-item anxiety scale using the same four-point ordinal scale as the PHQ-9 [52]. Scores range from 0 to 21. We defined possible incidences of generalised anxiety disorders using an established cut-off (scores ≥ 10) in the main analysis and continuous symptom score for the secondary analysis. This cut-off has a sensitivity of 89% and specificity of 82% [52].

### Confounding variables

We constructed a directed acyclic graph (DAG) of the proposed causal assumptions between exposure, outcome, and confounding variables that informed our analysis (Additional file 1, Figure 1). Possible confounding variables for this analysis include the following: age, sex, socioeconomic position (household income of < £18,000, £18,000 to £30,999, £31,000 to £51,999, £52,000 to £100,000, and > £100,0000), baseline mental health symptoms, smoking status (current, former, or never), total physical activity (total daily minutes spent walking and in moderate or vigorous activity from the International Physical Activity Questionnaire (IPAQ)), education level (degree, A/AS-level, O-level/GCSE, CSE, NVQ/HND/HNC, other qualifications, none), parental depression, chronic illness (self-reported yes or no), and diet (pieces of fruit and vegetable per day).

Our DAG indicated that adiposity (body fat percentage) could be on the causal pathway between fitness and common mental health symptoms. To avoid over-adjustment, we only include body fat as a confounding variable in the sensitivity analysis. Other variables used in the sensitivity analyses include a self-reported indicator of having visited a doctor or psychiatrist for depression, nerves, or anxiety in the past to identify participants with a history of common mental disorders.

### Analysis

Descriptive variables include means and standard deviations for normally distributed variables and medians and interquartile ranges for non-normally distributed variables.

#### Main analysis

The main analysis consists of two main components. The first aimed to determine longitudinal associations between individual domains of CRF, grip strength, and common mental health symptoms and disorders (aim 1). The second part investigated longitudinal associations between combined fitness and the incidence of common mental disorders (aim 2).

Firstly, we used logistic regression models with depression and anxiety incidence as outcomes and grip strength and CRF as categorical exposures, in separate models. We ran crude and fully adjusted iterations of all models. Secondly, the analysis of the combined role of CRF and grip strength derived logistic regression models with the same mental health outcomes and adjustments as our initial longitudinal models but using combined fitness as the main categorical exposure. Combined fitness includes both CRF and grip strength, which were positively correlated (*r* = 0.40). We compared combinations of CRF and grip strength, using the high CRF and high grip strength group as the reference category.

#### Secondary and sensitivity analysis

In the secondary analysis, we examined cross-sectional associations between CRF and grip strength with common mental health symptoms at baseline (*n* = 491,278). We used negative binomial regression models as with symptom scores on a quasi-continuous scale. We used negative binomial regression to account for the high positive skew and over-dispersion of the symptom scores. We present the output of negative binomial regressions as a percentage change in symptom scores. High fitness was the reference category for both exposure models.

We also reran the first part of the main analysis using continuous exposure variables (per 1-MET for CRF and per 5 kg for grip strength). We then created age and sex-standardised z scores for each exposure and ran the same models with age- and sex-exposure as multiplicative interaction terms to examine possible interactions.

We also carried out several sensitivity analyses determined a priori to examine the robustness of our main findings and explore alternative explanations. These included rerunning the longitudinal models from part 1 of the main analysis and in separate models: (1) excluding participants with a self-reported history of depression or anxiety prior to the baseline to further reduce the risk of residual confounding from reverse causation, (2) including adiposity as a possible confounding variable to examine the alternative hypothesis that adiposity is not on the causal pathway, and (3) using lower thresholds for defining depression (PHQ ≥ 8) and anxiety (GAD-7 ≥ 8) disorders [53, 54].

We then performed an adjusted multivariate linear model with CRF and grip strength as continuous exposures and PHQ-9 and GAD-7 scores as continuous outcomes. This was to assess whether both exposures were associated with depression and anxiety outcomes independently by testing each exposure coefficient across the equations for both outcomes. We also calculated *e* values to assess the plausibility of unmeasured confounding affecting our findings [55]. The *e* value estimates the required strength of an unmeasured confounding variable that would nullify the observed associations between our exposure and outcomes, while accounting for all measured covariates [56].

We reran the first part of the main longitudinal analysis in a full cohort with imputed missing data to assess the risk selection bias within our longitudinal sample (*n* = 152,978). We used multiple imputation models with chained equations to estimate missing data, as detailed in Methodology 1 (Additional file 1).

## Results

### Participants

At baseline, there were 491,278 participants with complete CRF or grip strength data. For the adjusted longitudinal (main) analysis, 147,141 participants had complete data for grip strength and 22,667 for CRF. For the cross-sectional (secondary) analysis, there were 465,757 participants in the fully adjusted analysis for grip strength and 60,838 for CRF data. According to the PHQ-9 and GAD-7 scales, 9156 (5.99%) participants met the criteria for depression, 5282 (3.45%) for anxiety, and 11,295 (7.39) for either common mental disorder at the 7-year follow-up. Tables 1 and 2 (Additional file 1) respectively contain baseline characteristics of participants by the CRF group and by grip strength group.

**Table 1:** Longitudinal associations between CRF and grip strength as separate exposures and common mental disorders

		Common mental disorders													
		Crude							Adjusted						
			Depression		Anxiety		Depression or anxiety			Depression		Anxiety		Depression or anxiety	
	Fitness group	***N***	OR (95% CIs)	***P***	OR (95% CIs)	***P***	OR (95% CIs)	***P***	***N***	OR (95% CIs)	***P***	OR (95% CIs)	***P***	OR (95% CIs)	***P***
CRF	Low	23,399	1.671 (1.455, 1.919)	< 0.001	1.228 (1.027, 1.469)	0.024	1.529 (1.350, 1.732)	< 0.001	22,667	1.596 (1.378, 1.849)	< 0.001	1.230 (1.020, 1.483)	0.030	1.485 (1.301, 1.694)	< 0.001
	Medium		1.172 (1.020, 1.347)	0.025	1.072 (0.904, 1.270)	0.425	1.160 (1.027, 1.311)	0.017		1.154 (0.999, 1.334)	0.051	1.067 (0.894, 1.272)	0.427	1.141 (1.005, 1.297)	0.041
	High		Reference							Reference					
Grip strength	Low	152,853	1.538 (1.461, 1.619)	< 0.001	1.454 (1.360, 1.554)	< 0.001	1.486 (1.418, 1.556)	< 0.001	147,141	1.410 (1.335, 1.490)	< 0.001	1.380 (1.286, 1.480)	< 0.001	1.381 (1.315, 1.452)	< 0.001
	Medium		1.118 (1.061, 1.178)	< 0.001	1.123 (1.051, 1.201)	0.001	1.107 (1.056, 1.160)	< 0.001		1.126 (1.066, 1.189)	< 0.001	1.145 (1.068, 1.228)	< 0.001	1.116 (1.063, 1.172)	< 0.001
	High		Reference							Reference					

Adjusted for age, sex, deprivation, smoking status, baseline symptoms, total physical activity, education, parental depression, chronic illness, and diet

*OR* odds ratio, *CIs* confidence intervals, *CRF* cardiorespiratory fitness

**Table 2:** Longitudinal associations between combined fitness categories and common mental disorders

	Common mental disorders													
	Crude							Adjusted						
	Depression		Anxiety		Depression or anxiety			Depression		Anxiety		Depression or anxiety		
Fitness group (*n* in group)	***N***	OR (95% CIs)	***P***	OR (95% CIs)	***P***	OR (95% CIs)	***P***	***N***	OR (95% CIs)	***P***	OR (95% CIs)	***P***	OR (95% CIs)	***P***
Low both (7054)	23,330	2.099 (1.667 2.645)	< 0.001	1.518 (1.133, 2.036)	0.024	1.866 (1.519, 2.295)	< 0.001	22,605	1.981 (1.553, 2.527)	< 0.001	1.559 (1.148, 2.118)	0.004	1.814 (1.461, 2.252)	< 0.001
Medium both (7071)		1.389 (1.096, 1.758)	0.006	1.118 (0.833, 1.500)	0.459	1.278 (1.037, 1.574)	0.021		1.427 (1.117, 1.825)	0.004	1.209 (0.894, 1.637)	0.217	1.325 (1.067, 1.645)	0.011
High both (8601)		Reference							Reference					

Adjusted for age, sex, deprivation, smoking status, baseline symptoms, total physical activity, education, parental depression, chronic illness

*OR* odds ratio, *CIs* confidence intervals, *CRF* cardiorespiratory fitness

### Main analysis

Longitudinal models for CRF and grip strength as separate exposures with incident depression, anxiety, and depression or anxiety as outcome variables are presented in Table 1. Adjusted models indicate that low CRF was associated with a 1.596 (95% CIs, 1.378, 1.849 *p* <  0.001) and medium CRF was associated with a 1.154 (95% CIs, 0.999, 1.334, *p* = 0.051) increase in the odds of depression compared with the high CRF group. Low CRF was associated with a 1.230 (95% CIs, 1.020, 1.483, *p* = 0.030) increase in the odds of anxiety compared to high CRF. Low CRF was associated with a 1.485 (95% CIs, 1.301, 1.694, *p* <  0.001) and medium CRF with a 1.141 (95% CIs, 1.005, 1.297, *p* = 0.041) increase in the odds of either common mental disorder compared to high CRF.

Low grip strength was associated with a 1.410 (95% CIs, 1.355, 1.490, *p* <  0.001) and medium with a 1.126 (95% CIs, 1.066, 1.189, *p* <  0.001) increase in the odds of depression compared with high grip strength. Compared with high, low grip strength was associated with a 1.380 (95% CIs, 1.286, 1.480, *p* <  0.001) and medium with a 1.145 (95% CIs, 1.068, 1.228, *p* <  0.001) increase in the odds of anxiety. Low grip strength was associated with 1.381 (95% CIs, 1.315, 1.452, *p* <  0.001) and medium with 1.116% (95% CIs, 1.063, 1.172, *p* <  0.001) higher odds of depression or anxiety.

Table 2 shows the results from the combined fitness exposures of CRF and grip strength. Compared to the high fitness group (high CRF *and* grip strength), low fitness (low CRF *and* low strength) was associated with 1.981 (95% CIs, 1.553, 2.527, *p* <  0.001) and medium with 1.427 (95% CIs, 1.117, 1.825, *p* = 0.004) higher odds of depression. Low fitness was associated with 1.599 (95% CIs, 1.148, 2.118, *p* = 0.004) higher odds of an anxiety disorder, compared with high fitness. Low fitness was associated with a 1.814 (95% CIs, 1.461, 2.252, *p* <  0.001) and medium with a 1.325 (95% CIs, 1.067, 1.645, *p* = 0.011) increase in the odds of depression or an anxiety disorder, compared with high combined fitness.

### Secondary analysis and sensitivity analysis

The fully adjusted cross-sectional analysis examining associations between CRF and common mental health symptoms included 63,372 participants. The models suggest a biological gradient with the medium CRF group associated with a 7% higher symptom score (95% CIs, 4.4%, 9.6%, *p* <  0.001) and low CRF associated with 17.5% higher symptom score (95% CIs, 14.6%, 20.6%, *p* <  0.001) compared to the high CRF group. A similar association was observed for grip strength: compared to the high grip strength group, the medium group had 8.6% (95% CIs, 7.7%, 9.6%, *p* <  0.001) higher scores and the low group had 26.8% (95% CIs, 25.7%, 27.9%, *p* <  0.001) higher scores.

Fully adjusted longitudinal models with a continuous exposure suggest each 1 MET increase in CRF was associated with a 2.4% lower depression score (95% CI, − 3%, − 1.8%, *p* <  0.001) and a 1.22% lower anxiety score (95% CI, − 2%, − 0.3%, *p* <  0.001). Each 5 kg increase in grip strength was associated with a 4.4% lower depression score (95% CI, − 4.8%, − 3.9%, *p* <  0.001) and a 4.8% lower anxiety score (95% CI, − 5.4%, − 4.2%, *p* <  0.001).

There was some evidence of an interaction between grip strength and sex (*p* = 0.018) and grip strength and age (*p* = 0.001) for anxiety disorders. For men, each 5 kg increase in grip strength was associated with reduced odds of an anxiety disorder (OR = 0.901, 95% CI, 0.855, 0.950, *p* <  0.001); for women, this reduction was greater (OR = 0.840, 95% CI, 0.809, 0.873, *p* <  0.001). In those aged < 54, a 5-kg increase in grip strength was associated with 12.5% reduction in odds of anxiety (OR = 0.875, 95% CI, 0.826, 0.927, *p* <  0.001), ages ≥ 54 to 65 with 22.8% reduced odds (OR = 0.772, 95% CI, 0.711, 0.837, *p* <  0.001), and those ≥ 65 with 27.3% reduced odds (OR = 0.727, 95% CI, 0.623, 0.850, *p* <  0.001).

The results of our sensitivity analyses (see Additional file 1, Tables 3 to 6) indicate no substantial differences from our main findings. The results from our analyses in a fully imputed cohort were consistent with the findings in the main analysis (Additional file 1, Table 6). The *e* values estimate the required strength of an unmeasured confounding variable to nullify the observed associations between our exposure and outcomes. For longitudinal models of the association between low CRF (vs. high) with depression, the *e* value was 2.57 (CI = 2.1), with anxiety was 1.76 (CI = 1.16), and depression or anxiety was 2.33 (CI = 1.93). The ORs for observed confounding variables in these models ranged between 0.65 and 2.07. For associations between low grip strength (vs. high) with depression, the *e* value was 2.17 (CI = 2), with anxiety it was 2.1 (CI = 1.89), and for depression or anxiety, it was 2.11 (CI = 1.96). Observed confounding variables in these models had ORs ranging from 0.64 to 2.16. For low combined fitness (vs. high), the *e* value was 3.38 (CI = 2.48) for depression, 2.49 (CI = 1.56) for anxiety, and 3.03 (CI = 2.28) for depression or anxiety. Observed confounding variables in these models had ORs ranging from 0.60 to 2.06.

## Discussion

### Main findings

To the best of our knowledge, this is the first prospective study to examine associations between individual and combined CRF and grip strength with the incidence of common mental disorders in the general population. Low combined fitness (low CRF and low grip strength) was associated with 1.8 times the odds of a common mental disorder compared to high combined fitness, with the medium combined fitness group having 1.3 times the odds. Low combined fitness was associated with 2.0 and 1.6 times higher the odds of depression and anxiety disorders, respectively. When looking at CRF and grip strength as separate exposures, the effect sizes were smaller. We found that compared with high CRF, low CRF was associated with 1.5 times higher odds of a common mental disorder incidence and low grip strength with 1.4 times higher odds. There was some evidence of a dose-response relationship between fitness and the incidence of common mental disorders. We also found that associations with grip strength and anxiety disorders had higher odds ratios for women than men, and for older than younger adults.

These findings were robust to a series of sensitivity analyses. The findings are unlikely to be nullified by an unmeasured confounding variable according to the *e* values. For example, the *e* value for combined fitness groups indicates an unmeasured confounding variable would require an association of at least OR = 3.0 with fitness and common mental disorders to nullify the observed associations. The observed confounding variables had ORs ranging from 0.6 to 2.1, suggesting an OR of 3 for an unmeasured confounding variable is unlikely. Our results were also consistent in a full cohort with imputed missing data.

Our study is novel in demonstrating that the combination of low CRF and grip strength is associated with a higher risk of common mental disorders than either type of fitness alone. This finding highlights the importance of focusing on multiple components of fitness and their associations with mental health. Findings for individual exposures align with results from recent meta-analyses of 4 studies that suggested low CRF was associated with an increased risk of common mental disorders of similar effect size (HR = 1.47, 95% CI 1.23 to 1.76), which also suggested a dose-response relationship [28]. These findings build on previous prospective studies in smaller samples [34–37] to suggest that low grip strength is a possible risk factor for common mental disorders in adult men and women.

### Strengths and limitations

This study benefitted from a large sample size and a 7-year follow-up period. It included objective measures of fitness administered by trained staff using validated protocols. The prospective study design, dose-response relationship, consistent results from several sensitivity analyses (including the removal of participants with a history of depression or anxiety), and multiple imputation models, suggest possible biases such as attrition bias, reverse causation, or unmeasured confounding are unlikely to explain our results. The use of DAGs to inform our analysis also improved our ability to estimate causal effects.

The study also had several limitations. We estimated CRF from a submaximal exercise test rather than a gold standard maximal exercise with gas analysis [57]. However, these tests are prohibitively expensive in large samples and we are aware of only one previous study (*n* = 1575) in this area using these gold standard exercise protocols [58]. Exercise testing was a late addition to Biobank data collection protocols in 2009 and only available for ~ 14% of participants. However, these participants are representative of the wider Biobank sample in terms of sociodemographic and biological characteristics [47]. Despite the low response rate of 5.5% in obtaining the final Biobank sample, participants are comparable with the general population on several sociodemographic and physical health factors [44, 59].

The observational nature of this study also includes the risk of unmeasured confounding biasing our results. The *e* values suggest it is unlikely that a single unmeasured confounding variable would explain our main findings. However, multiple unmeasured variables together might explain the associations we found. There may also be some measurement error in the self-reported scales used to record common mental health symptoms compared with clinical diagnoses. However, these scales allowed us to assess symptoms in people who do not seek treatment or are without a formal mental health diagnosis and are extensively validated. It was also not possible to reliably estimate common mental disorder incidence at baseline, only symptoms.

### Implications and future research

Aerobic and resistance training improves different aspects of physical fitness and randomised controlled trials have found that both types of training can each reduce common mental health symptoms [5–11]. At a population level, improving multiple aspects of fitness could contribute towards a reduction in the incidence of common mental disorders. While broadly increasing physical activity will be beneficial [12, 13], structured aerobic and resistance exercises with sufficient intensity to improve fitness may have a greater effect on risk reduction. These combined approaches may also have additive benefits for reducing the physical health risks [39–42] associated with common mental disorders.

It is possible to modify fitness through simple, low-cost physical activity interventions, including in people with common mental health symptoms [60]. Substantial improvements in fitness are possible within a short timeframe. For example, one study in previously untrained older adults suggested that 3 weeks of regular aerobic exercise was sufficient to improve CRF by 31%, which continued to increase with further training [61]. Our data suggest that a 31% increase would equate to moving from low to medium CRF and reduce the odds of a common mental disorder by 14.1%. Similar improvements in grip strength over the same period would further reduce the odds of a common mental disorder by 32.5%.

Objective markers of physical fitness approximate habitual physical activity and could also be useful population-level indicators of mental health risk in their own right. Most studies of associations between physical activity and the incidence of common mental disorders use self-reported activity data [12, 13]. Our study suggests that objective markers of physical fitness could be useful indicators of mental health risk in the population, as they are for physical health risks [20, 21]. Meta-analyses suggest that high physical activity is associated with a 0.83 (95% CI, 0.79 to 0.88) and 0.74 (95% CI, 0.62 to 0.88) [12, 13] lower odds of a depression and anxiety disorders respectively. However, effect sizes for low (combined or individual) physical fitness appear larger in this study and previous meta-analyses [28]. Objective markers of fitness are increasingly recognised as stronger indicators of cardiovascular disease than physical activity [25, 62], and the same may be true for mental health.

The collection of population-level physical activity data will continue to play a foundational role in monitoring physical and mental health but remains challenging [63]. Physical fitness is a stable surrogate marker of habitual physical activity with objective, clearly defined outputs, such as oxygen uptake [18]. Fitness may also act through pathways independent of general physical activity [64, 65]. For example, measures of CRF can incorporate additional information by capturing the complex interplay between other relevant factors to mental health, such as genetics, body composition, and smoking [66]. Increased efforts to collect population-level physical fitness data could inform our understanding of mental health and the development of public health strategies.

## Conclusions

Low CRF and grip strength are both associated with an increased incidence of common mental disorders, and the combination of low CRF and low grip strength was associated with the highest level of risk. Physical fitness could be an objectively measurable indicator and a modifiable risk factor for common mental disorders in the population. Public health approaches to improve physical fitness through combined aerobic and resistance activities could reduce the incidence of common mental disorders and improve physical health outcomes for people with mental health symptoms.

## Supplementary information

**Additional file 1.** Additional details on the methodology (Figure 1 and Methodology 1), baseline participant characteristics (Tables 1 and 2), sensitivity analyses results (Tables 3 to 6), and STROBE statement.

## Data Availability

All bona fide researchers can apply to access and use the UK Biobank resource.

## Abbreviations

CRF: Cardiorespiratory fitness
