# Supplementary material for: Individual and combined associations between cardiorespiratory fitness and grip strength with common mental disorders: a prospective cohort study in the UK Biobank
Source: BMC Med. 2020 Nov 11;18:303. doi: 10.1186/s12916-020-01782-9 (PMC7656705; doi:10.1186/s12916-020-01782-9)
Supplement: Supplementary file 1 — Additional file 1. Additional details on the methodology (Figure 1 and Methodology 1), baseline participant characteristics (Tables 1 and 2), sensitivity analyses results (Tables 3 to 6), and STROBE statement. [file 12916_2020_1782_MOESM1_ESM.docx]

**Additional file 1**


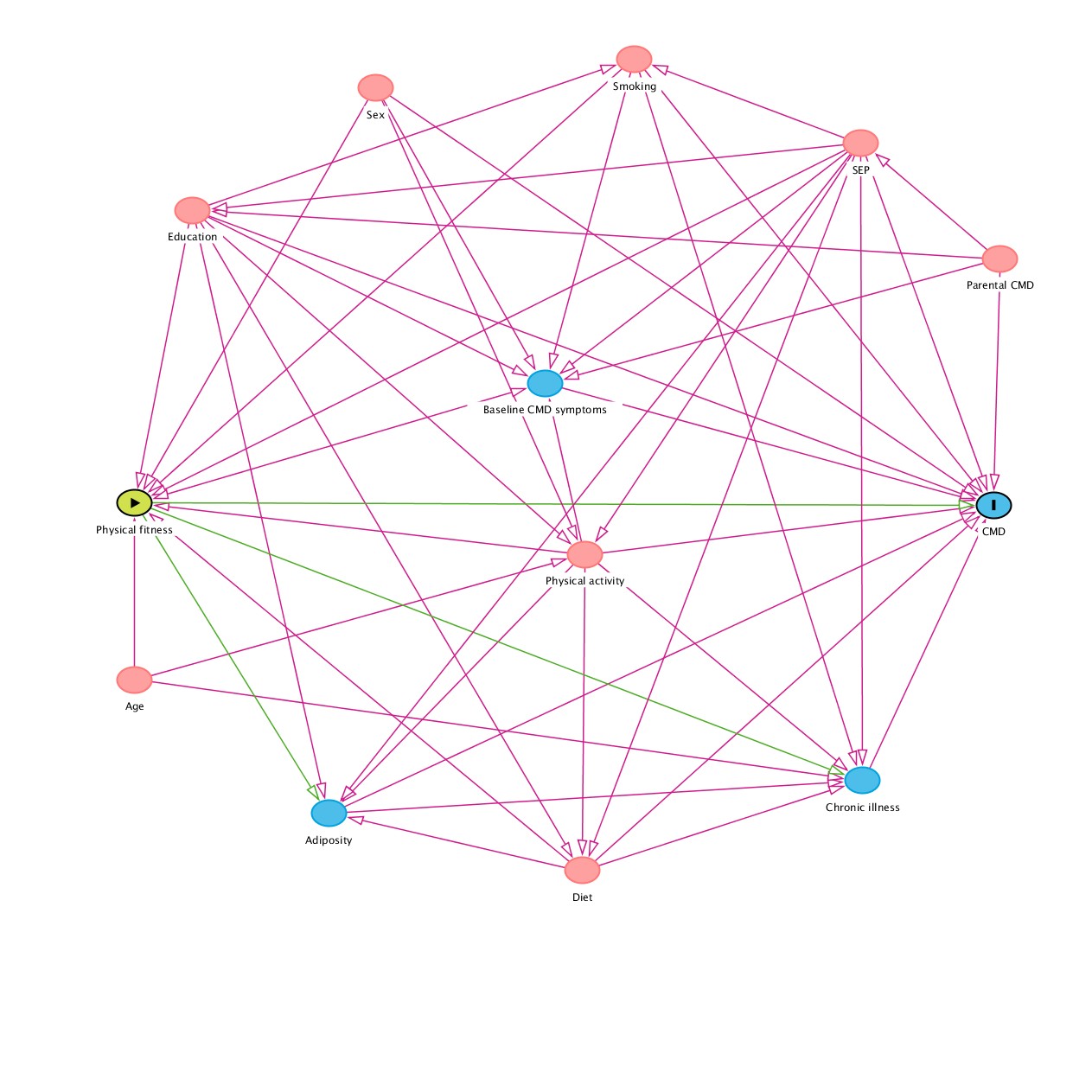
**Figure 1. DAG of proposed causal associations between fitness, common mental disorders, and covariates**

*CMD = common mental disorder; SEP = socioeconomic position*

**Methodology 1. Multiple imputation models**

We ran multiple imputation models by chained equations to generate 50 datasets for 152,978 in the longitudinal analysis. The method uses separate distributions per imputed variable. Non-normally distributed continuous variables were imputed using predictive mean matching. The results from all 50 datasets are pooled together with corrected standard errors according to Rubin’s rule. The multiple imputation model included all variables used in our analysis for the exposure, outcome, and covariates, including those in the sensitivity analysis.

**Table 1. Baseline participant characteristics by CRF groupings**

|  |  |  | CRF (n = 63,372) | | |
| --- | --- | --- | --- | --- | --- |
| Variable |  | All | Low | Medium | High |
| N |  | 491,278 | 17,952 (28.33) | 20,257 (31.97) | 25,163 (39.71) |
| Sex (%) | Women | 267,392 (54.43) | 9621 (53.59) | 10991 (54.26) | 13409 (53.29) |
|  | Men | 223,886 (45.57) | 8331 (46.41) | 9266 (45.74) | 11754 (46.71) |
| Age | Mean (SD) | 56.559 (8.090) | 57.013 (7.837) | 56.63 (8.10) | 56.20 (8.34) |
| Ethnicity | White | 463630 (94.72) | 15221 (85.18) | 18524 (91.88) | 23852 (95.13) |
|  | Mixed | 2884 (0.59) | 174 (0.97) | 180 (0.89) | 202 (0.81) |
|  | South Asian | 7732 (1.58) | 933 (5.22) | 635 (3.15) | 359 (1.43) |
|  | Black | 1497 (0.31) | 1086 (6.08) | 484 (2.40) | 274 (1.09) |
|  | Chinese | 9347 (1.91) | 63 (0.35) | 81 (0.40) | 126 (0.50) |
|  | Other | 4367 (0.89) | 392 (2.19) | 257 (1.27) | 260 (1.04) |
| Household income | Less than 18,000 | 95316 (22.86) | 3807 (25.19) | 3340 (19.02) | 3480 (15.62) |
|  | 18,000 to 30,999 | 106335 (25.50) | 4164 (27.56) | 4399 (25.05) | 5035 (22.60) |
|  | 31,000 to 51,999 | 108634 (26.06) | 3844 (25.44) | 4773 (27.18) | 5844 (26.23) |
|  | 52,000 to 100,000 | 84405 (20.24) | 2633 (17.42) | 3881 (22.10) | 5692 (25.55) |
|  | Greater than 100,000 | 22248 (5.34) | 663 (4.39) | 1165 (6.64) | 2229 (10.00) |
| Education level | None | 83697 (17.35) | 2701 (13.47) | 2502 (10.00) | 8273 (13.18) |
|  | College or University degree | 157403 (32.63) | 7090 (35.36) | 10741 (42.94) | 22925 (36.52) |
|  | A levels/AS levels or equivalent | 54196 (11.24) | 2370 (11.82) | 3096 (12.38) | 7489 (11.93) |
|  | O levels/GCSEs or equivalent | 103278 (21.41) | 4385 (21.87) | 4881 (19.51) | 13298 (21.18) |
|  | CSEs or equivalent | 26339 (5.46) | 1124 (5.61) | 1161 (4.64) | 3462 (5.51) |
|  | NVQ or HND or HNC or equivalent | 32125 (6.66) | 1363 (6.80) | 1380 (5.52) | 4096 (6.52) |
|  | Other professional qualifications e.g., nursing, teaching | 25310 (5.25) | 1020 (5.09) | 1251 (5.00) | 3232 (5.15) |
| Parental depression | No | 434749 (91.14) | 15991 (91.73) | 17976 (91.07) | 22204 (90.40) |
|  | Yes | 42248 (8.86) | 1441 (8.27) | 1762 (8.93) | 2357 (9.60) |
| Chronic illness or disability | No | 322634 (65.80) | 11236 (62.76) | 14242 (70.39) | 18432 (73.29) |
|  | Yes | 156670 (31.95) | 6169 (34.46) | 5545 (27.40) | 6294 (25.03) |
| Body fat % | mean (SD) | 31.458 (8.545) | 34.188 (8.425) | 31.506 (7.958) | 28.696 (7.853) |
| Grip strength per 5kg | mean (SD) | 6.121 (2.205) | 5.84 (2.105) | 5.978 (2.117) | 6.149 (2.118) |
| CRF, METs | mean (SD) | 9.951 (2.971) | 7.171 (1.438) | 9.311 (1.519) | 12.452 (2.606) |
| Daily minutes of physical activity | mean (SD) | 125.429 (145.591) | 114.057 (139.592) | 127.198 (137.176) | 140.215 (139.356) |
| Daily pieces of fruit and vegetables | mean (SD) | 4.898 (2.751) | 4.793 (2.835) | 4.876 (2.822) | 5.022 (2.747) |
| Smoking status | Never | 35424 (56.08) | 10415 (58.25) | 11304 (55.97) | 13705 (54.62) |
|  | Previous | 21964 (34.77) | 6023 (33.68) | 7032 (34.82) | 8909 (35.51) |
|  | Current | 5779 (9.15) | 1443 (8.07) | 1859 (9.21) | 2477 (9.87) |

**Table 2. Baseline participant characteristics by CRF groupings**

|  |  |  | Grip strength (n = 491,108) | | |
| --- | --- | --- | --- | --- | --- |
| Variable |  | All | Low | Medium | High |
| N (%) |  | 491,278 | 151,300 (30.81) | 165,629 (33.73) | 174,179 (35.47) |
| Sex (%) | Women | 267,392 (54.43) | 82151 (54.30) | 89563 (54.07) | 95591 (54.88) |
|  | Men | 223,886 (45.57) | 69149 (45.70) | 76066 (45.93) | 78588 (45.12) |
| Age | Mean (SD) | 56.559 (8.090) | 56.695 (8.023) | 56.888 (7.853) | 56.12 (8.344) |
| Ethnicity | White | 463630 (94.72) | 138717 (92.06) | 157700 (95.52) | 167213  (96.27) |
|  | Mixed | 2884 (0.59) | 922 (0.61) | 878 (0.53) | 1084 (0.62) |
|  | South Asian | 7732 (1.58) | 2465 (1.64) | 2296 (1.39) | 2971 (1.71) |
|  | Black | 1497 (0.31) | 665 (0.44) | 505 (0.31) | 327 (0.19) |
|  | Chinese | 9347 (1.91) | 5850 (3.88) | 2421 (1.47) | 1076 (0.62) |
|  | Other | 4367 (0.89) | 2054 (1.36) | 1301 (0.79) | 1012 (0.58) |
| Household income | Less than 18,000 | 95316 (22.86) | 36406  (29.05) | 31196 (22.10) | 27714 (18.41) |
|  | 18,000 to 30,999 | 106335 (25.50) | 32921 (26.27) | 36302 (25.72) | 37112 (24.66) |
|  | 31,000 to 51,999 | 108634 (26.06) | 29752 (23.74) | 37150 (26.32) | 41732 (27.73) |
|  | 52,000 to 100,000 | 84405 (20.24) | 21012 (16.77) | 28828 (20.43) | 34565 (22.97) |
|  | Greater than 100,000 | 22248 (5.34) | 5214 (4.16) | 7656 (5.42) | 9378 (6.23) |
| Education level | None | 83697 (17.35) | 31784 (21.46) | 27705  (17.02) | 24208  (14.12) |
|  | College or University degree | 157403 (32.63) | 43184 (29.16) | 53043  (32.59) | 61176  (35.67) |
|  | A levels/AS levels or equivalent | 54196 (11.24) | 15741 (10.63) | 18360  (11.28) | 20095  (11.72) |
|  | O levels/GCSEs or equivalent | 103278 (21.41) | 31392 (21.19) | 35533  (21.83) | 36353  (21.20) |
|  | CSEs or equivalent | 26339 (5.46) | 8713 (5.88) | 8835 (5.43) | 8791 (5.13) |
|  | NVQ or HND or HNC or equivalent | 32125 (6.66) | 9750 (6.58) | 10705 (6.58) | 11670 (6.80) |
|  | Other professional qualifications e.g., nursing, teaching | 25310 (5.25) | 7548 (5.10) | 8562 (5.26) | 9200 (5.36) |
| Parental depression | No | 434749 (91.14) | 132770 (90.67) | 146749 (91.11) | 155230 (91.58) |
|  | Yes | 42248 (8.86) | 13667 (9.33) | 14317 (8.89) | 14264 (8.42) |
| Chronic illness or disability | No | 322634 (65.80) | 86103 (57.04) | 112045 (67.74) | 124486 (71.55) |
|  | Yes | 156670 (31.95) | 61022 (40.43) | 49588 (29.98) | 46060 (26.47) |
| Body fat % | mean (SD) | 31.458 (8.545) | 32.12 (8.679) | 31.282 (8.522) | 31.054 (8.421) |
| Grip strength per 5kg | mean (SD) | 6.121 (2.205) | 4.498 (1.602) | 6.076 (1.709) | 7.575 (2.075) |
| CRF, METs | mean (SD) | 9.951 (2.971) | 9.722 (2.887) | 9.966 (3.002) | 10.191 (3.002) |
| Daily minutes of physical activity | mean (SD) | 125.429 (145.591) | 113.267 (141.988) | 126.639 (145.651) | 133.927 (147.834) |
| Daily pieces of fruit and vegetables | mean (SD) | 4.898 (2.751) | 4.813 (2.925) | 4.883 (2.691) | 4.963 (2.676) |
| Smoking status | Never | 83297 (55.32) | 83297 (55.32) | 90837 (55.03) | 93694 (53.96) |
|  | Previous | 50129 (33.29) | 50129 (33.29) | 57063 (34.57) | 62440 (35.96) |
|  | Current | 17153 (11.39) | 17153 (11.39) | 17159 (10.40) | 17504 (10.08) |

|  | | **Common mental health disorders** | | | | | | |
| --- | --- | --- | --- | --- | --- | --- | --- | --- |
|  |  | **Adjusted** | | | | | | |
|  |  |  | **Depression** | | **Anxiety** | | **Depression or anxiety** | |
|  | **Fitness group** | **N** | **OR (95% CIs)** | **P** | **OR (95% CIs)** | **P** | **OR (95% CIs)** | **P** |
| CRF | Low | 15,363 | 1.75 (1.143, 1.905) | 0.003 | 1.339 (0.964, 1.858) | 0.081 | 1.522 (1.220, 1.900) | < 0.001 |
|  | Medium |  | 1.371 (1.082, 1.736) | 0.009 | 1.120 (0.822, 1.153) | 0.471 | 1.308 (1.062, 1.611) | 0.011 |
|  | High |  | Reference | | | | | |
| Grip strength | Low | 97,880 | 1.409 (1.285, 1.545) | < 0.001 | 1.358 (1.196, 1.115) | < 0.001 | 1.393 (1.284, 1.514) | < 0.001 |
|  | Medium |  | 1.110 (1.013, 1.215) | 0.0024 | 1.125 (1.115, 1.417) | < 0.001 | 1.134 (1.046, 1.229) | 0.002 |
|  | High |  | Reference | | | | | |

**Table 3. Longitudinal models with participants with a history of depression or anxiety excluded**

**Table 4. Longitudinal models adiposity included as a covariate**

|  | | **Common mental health disorders** | | | | | | | |
| --- | --- | --- | --- | --- | --- | --- | --- | --- | --- |
|  |  | **Adjusted** | | | | | | |  |
|  |  |  | **Depression** | | **Anxiety** | | **Depression or anxiety** | |  |
|  | **Fitness group** | **N** | **OR (95% CIs)** | **P** | **OR (95% CIs)** | **P** | **OR (95% CIs)** | **P** |  |
| CRF | Low | 23,399 | 1.328 (1.131, 1.559) | 0.001 | 1.180 (0.961, 1.446) | 0.115 | 1.281 (1.110, 1.480) | 0.001 |  |
|  | Medium |  | 1.044 (0.900, 1.213) | 0.566 | 1.050 (0.876, 1.206) | 0.596 | 1.056 (0.925, 1.203) | 0.423 |  |
|  | High |  | Reference | | | | | |  |
| Grip strength | Low | 152,853 | 1.366 (1.296, 1.444) | < 0.001 | 1.356 (1.262, 1.456) | < 0.001 | 1.343 (1.278, 1.413) | < 0.001 |  |
|  | Medium |  | 1.112 (1.063, 1.444) | < 0.001 | 1.142 (1.064, 1.224) | < 0.001 | 1.112 (1.058, 1.168) | < 0.001 |  |
|  | High |  | Reference | | | | | |  |

|  | | **Common mental health disorders** | | | | | | |
| --- | --- | --- | --- | --- | --- | --- | --- | --- |
|  |  | **Adjusted** | | | | | | |
|  |  |  | **Depression** | | **Anxiety** | | **Depression or anxiety** | |
|  | **Fitness group** | **N** | **OR (95% CIs)** | **P** | **OR (95% CIs)** | **P** | **OR (95% CIs)** | **P** |
| CRF | Low | 23,399 | 1.475 (1.309, 1.660) | < 0.001 | 1.351 (1.159, 1.570) | < 0.001 | 1.485 (1.302, 1.695) | < 0.001 |
|  | Medium |  | 1.195 (1.067, 1.337) | 0.002 | 1.157 (1.002, 1.337) | 0.046 | 1.141 (1.005, 1.296) | 0.041 |
|  | High |  | Reference | | | | | |
| Grip strength | Low | 152,853 | 1.354 (1.286, 1.405) | < 0.001 | 1.402 (1.322, 1.486) | < 0.001 | 1.381 (1.315, 1.452) | < 0.001 |
|  | Medium |  | 1.116 (1.068, 1.165) | < 0.001 | 1.140 (1.076, 1.207) | < 0.001 | 1.112 (1.061, 1.173) | < 0.001 |
|  | High |  | Reference | | | | | |

**Table 5. Longitudinal models lower thresholds for defining depression (PHQ ≥8) and anxiety (GAD-7 ≥8) incidence**

| **Fully imputed** | | **Common mental health disorders** | | | | | | |
| --- | --- | --- | --- | --- | --- | --- | --- | --- |
|  |  | **Adjusted** | | | | | | |
|  |  |  | **Depression** | | **Anxiety** | | **Depression or anxiety** | |
|  | **Fitness group** | **N** | **OR (95% CIs)** | **P** | **OR (95% CIs)** | **P** | **OR (95% CIs)** | **P** |
| CRF | Low | 152,978 | 1.628 (1.436, 1.846) | < 0.001 | 1.256 (1.072, 1.472) | < 0.001 | 1.489 (1.332, 1.664) | < 0.001 |
|  | Medium |  | 1.252 (1.133, 1.384) | < 0.001 | 1.102 (0.982, 1.238) | 0.096 | 1.198 (1.096, 1.307) | < 0.001 |
|  | High |  | Reference | | | | | |
| Grip strength | Low | 152,978 | 1.385 (1.312, 1.460) | < 0.001 | 1.356 (1.266, 1.452) | < 0.001 | 1.359 (1.294, 1.426) | < 0.001 |
|  | Medium |  | 1.122 (1.064, 1.183) | < 0.001 | 1.140 (1.065, 1.220) | < 0.001 | 1.115 (1.063, 1.170) | < 0.001 |
|  | High |  | Reference | | | | | |

**Multivariate models**

The results of the multivariate linear model indicate that CRF is independently associated with both PHQ-9 and GAD-7 outcomes, following a test of the coefficient across both equations (F(2, 23,399) = 35.36, *p* < 0.001). Grip strength was also independently associated with each outcome (F(2, 152,853) = 207.09, *p* < 0.001).

**Table 6. Longitudinal models in full cohort with imputed missing data**

**STROBE Statement**

|  | Item No | Recommendation | Page No |
| --- | --- | --- | --- |
| **Title and abstract** | 1 | (*a*) Indicate the study’s design with a commonly used term in the title or the abstract | 1 and 2 |
|  |  | (*b*) Provide in the abstract an informative and balanced summary of what was done and what was found |  |
| Introduction | | | |
| Background/rationale | 2 | Explain the scientific background and rationale for the investigation being reported | 4 and 5 |
| Objectives | 3 | State specific objectives, including any prespecified hypotheses | 5 |
| Methods | | | |
| Study design | 4 | Present key elements of study design early in the paper | 5 |
| Setting | 5 | Describe the setting, locations, and relevant dates, including periods of recruitment, exposure, follow-up, and data collection | 5 |
| Participants | 6 | (*a*) Give the eligibility criteria, and the sources and methods of selection of participants. Describe methods of follow-up | 5 |
|  |  | (*b*) For matched studies, give matching criteria and number of exposed and unexposed |  |
| Variables | 7 | Clearly define all outcomes, exposures, predictors, potential confounders, and effect modifiers. Give diagnostic criteria, if applicable | 5 to 7 |
| Data sources/ measurement | 8* | For each variable of interest, give sources of data and details of methods of assessment (measurement). Describe comparability of assessment methods if there is more than one group | 5 to 7 |
| Bias | 9 | Describe any efforts to address potential sources of bias | 7 and 8 |
| Study size | 10 | Explain how the study size was arrived at | 5 |
| Quantitative variables | 11 | Explain how quantitative variables were handled in the analyses. If applicable, describe which groupings were chosen and why | 7 and 8 |
| Statistical methods | 12 | (*a*) Describe all statistical methods, including those used to control for confounding | 7 and 8 |
|  |  | (*b*) Describe any methods used to examine subgroups and interactions |  |
|  |  | (*c*) Explain how missing data were addressed |  |
|  |  | (*d*) If applicable, explain how loss to follow-up was addressed |  |
|  |  | (*e*) Describe any sensitivity analyses |  |
| Results | | |  |
| Participants | 13* | (a) Report numbers of individuals at each stage of study—eg numbers potentially eligible, examined for eligibility, confirmed eligible, included in the study, completing follow-up, and analysed | 5 and 8 to 9 |
|  |  | (b) Give reasons for non-participation at each stage |  |
|  |  | (c) Consider use of a flow diagram |  |
| Descriptive data | 14* | (a) Give characteristics of study participants (eg demographic, clinical, social) and information on exposures and potential confounders | Supplementary |
|  |  | (b) Indicate number of participants with missing data for each variable of interest |  |
|  |  | (c) Summarise follow-up time (eg, average and total amount) |  |
| Outcome data | 15* | Report numbers of outcome events or summary measures over time | 9 |

| Main results | 16 | (*a*) Give unadjusted estimates and, if applicable, confounder-adjusted estimates and their precision (eg, 95% confidence interval). Make clear which confounders were adjusted for and why they were included | 9 to 10 |
| --- | --- | --- | --- |
|  |  | (*b*) Report category boundaries when continuous variables were categorized |  |
|  |  | (*c*) If relevant, consider translating estimates of relative risk into absolute risk for a meaningful time period |  |
| Other analyses | 17 | Report other analyses done—eg analyses of subgroups and interactions, and sensitivity analyses | 10 |
| Discussion | | | |
| Key results | 18 | Summarise key results with reference to study objectives | 11 |
| Limitations | 19 | Discuss limitations of the study, taking into account sources of potential bias or imprecision. Discuss both direction and magnitude of any potential bias | 11 and 12 |
| Interpretation | 20 | Give a cautious overall interpretation of results considering objectives, limitations, multiplicity of analyses, results from similar studies, and other relevant evidence | 11 to 13 |
| Generalisability | 21 | Discuss the generalisability (external validity) of the study results | 11 to 13 |
| Other information | | | |
| Funding | 22 | Give the source of funding and the role of the funders for the present study and, if applicable, for the original study on which the present article is based | 14 |

*Give information separately for exposed and unexposed groups.
